# Prevalence of malaria and scrub typhus co-infection in febrile patients: a systematic review and meta-analysis

**DOI:** 10.1186/s13071-021-04969-y

**Published:** 2021-09-14

**Authors:** Polrat Wilairatana, Saruda Kuraeiad, Pongruj Rattaprasert, Manas Kotepui

**Affiliations:** 1 Department of Clinical Tropical Medicine, Faculty of Tropical Medicine, Mahidol University, Bangkok, 10400 Thailand; 2 Department of Medical Technology, School of Allied Health Sciences, Walailak University, Tha Sala, Nakhon Si Thammarat, 80160 Thailand; 3 Department of Protozoology, Faculty of Tropical Medicine, Mahidol University, Bangkok, 10400 Thailand

**Keywords:** Scrub typhus, *Orientia tsutsugamushi*, Malaria, *Plasmodium* spp., Co-infection

## Abstract

**Background:**

Little information is available about malaria and scrub typhus co-infection. This study aimed to investigate the pooled prevalence of malaria and scrub typhus co-infection in febrile patients. Further, it aimed to estimate the prevalence of scrub typhus infection among patients with malaria and the odds of co-infection. This will aid the diagnosis and management of co-infected patients in endemic areas.

**Methods:**

We searched for relevant studies in three databases: PubMed, Scopus, and Web of Science. We assessed the quality of the included studies using the Joanna Briggs Institute checklist for analytical cross-sectional studies. We estimated (1) the pooled prevalence of malaria and scrub typhus co-infection, (2) the pooled prevalence of scrub typhus infection in malaria-positive patients, and (3) the pooled odds of co-infection using the DerSimonian–Laird method for random-effects models. The study results and summary estimates were visualized on a forest plot as point estimates (effect size, prevalence) and 95% confidence intervals (CI). We assessed the heterogeneity of the studies by Cochrane Q or *I*^2^ statistics. We performed subgroup analyses of countries and scrub typhus diagnostic tests to explore the sources of heterogeneity of the included studies. We assessed publication bias if more than 10 studies were used to estimate the outcome. All data analyses were conducted using Stata version 14 (StataCorp, College Station, TX, USA).

**Results:**

Of the 542 studies retrieved from three databases, we included 14 meeting the inclusion criteria in the systematic review and meta-analysis. The pooled prevalence of malaria and scrub typhus co-infection (56 cases) among febrile patients (7920 cases) was 1% (95% CI: 0–1%, *I*^2^: 78.28%), while the pooled prevalence of scrub typhus infection (321 cases) in patients with malaria (1418 cases) was 21% (95% CI: 12–30%, *I*^2^: 98.15%). Subgroup analysis showed that the pooled prevalence of scrub typhus infection among patients with malaria in India was 8% (95% CI: 4–13%, *I*^2^: 85.87%, nine studies with 59/794 cases), while the pooled prevalence of scrub typhus infection among patients with malaria in Thailand was 35% (95% CI: 7–64%, *I*^2^: 98.9%, four studies with 262/624 cases). The co-infections did not occur by chance (*P* = 0.013, odds: 0.43, 95% CI: 0.22–0.84%, *I*^2^: 60.9%). In the sensitivity analysis, the pooled prevalence of malaria and scrub typhus co-infection among febrile patients was 0% (95% CI: 0–1%, *I*^2^: 59.91%).

**Conclusions:**

The present study showed the pooled prevalence and a significant association between malaria and scrub typhus. The results show the status of co-infection. Further research into co-infection in endemic areas is needed, in particular, to determine whether co-infection can accelerate disease progression or protect against severe disease.

**Graphical abstract:**

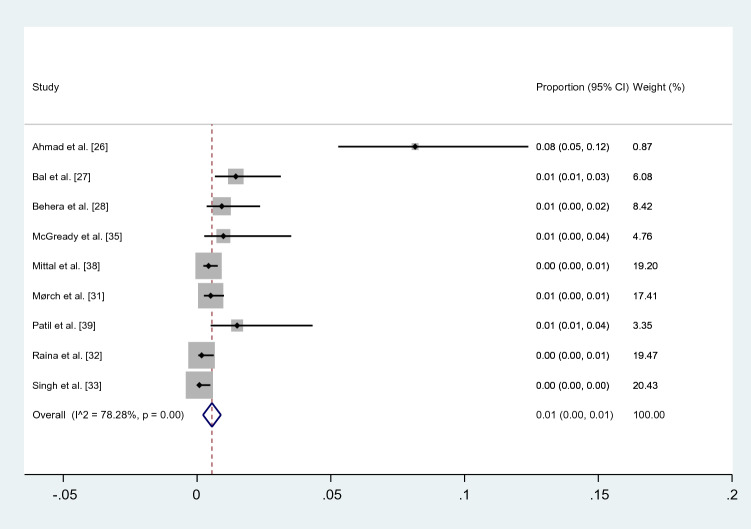

**Supplementary Information:**

The online version contains supplementary material available at 10.1186/s13071-021-04969-y.

## Background

Blood-feeding *Anopheles* mosquitoes transmit malaria by one of the six *Plasmodium* species, *P. falciparum*, *P. vivax*, *P. malariae*, *P. ovale curtisi*, *P. ovale wallikeri*, and *P. knowlesi*; however, some zoonotic *Plasmodium* species, such as *P. cynomolgi* and *P. simium*, have been associated with human cases of malaria [1–5]. In a high transmission area, malaria is a major cause of death in children under 5 years because they lack immunity against malaria parasites; malaria is less common in older children and adults because of partial immunity [6]. In a low transmission setting, symptomatic malaria may occur at all ages, particularly in semi-immune or non-immune people [7, 8]. In 2020, the World Health Organization (WHO) reported that the Southeast Asian region accounted for ~ 3% of the global malaria burden of 6.3 million, and malaria deaths reached ~ 9000 in 2019 [7]. Within the WHO South-East Asia Region, India had 86% of malaria deaths, while in the Greater Mekong Subregion, Cambodia (58%) and Myanmar (31%) reported the most malaria cases [7].

Scrub typhus, which causes an acute febrile illness, is caused by the Gram-negative obligate intracellular bacillus *Orientia tsutsugamushi.* It is transmitted via trombiculid mite chigger (larva) bites [9] and is associated with agricultural work and rural dwellings [10, 11]. Recent findings suggest that other *Orientia* species are related to scrub typhus, including “*Candidatus* Orientia chiloensis” [12] and “*Candidatus* Orientia chuto” [13, 14]. Scrub typhus is endemic to the Asian-Pacific area with a seroprevalence ranging from 9.3% to 27.9% (median 22.2% interquartile range [IQR] 18.6–25.7). Reported case-fatality rates are 12.2% in South India and 13.6% in northern Thailand [15]. Scrub typhus seropositivity has also been evidenced in Honduras [16] and the Peruvian Amazon [17], with a seroprevalence of 5.6% and 5.3%. A recent finding reported that cases of scrub typhus had been found in southern Chile [13, 14, 18]. Therefore, scrub typhus is not limited to the Tsutsugamushi Triangle.

Clinical signs and symptoms of scrub typhus are fever, headache, myalgia, cough, and gastrointestinal symptoms, with or without escharation [19]. A recent study from northern Vietnam reported that 70% of patients with scrub typhus had eschars, 60% had skin-conjunctiva congestion, and 44% had lymphadenopathy [20]. Untreated acute scrub typhus cases have a high risk of developing severe manifestations, including microangiopathy, septic shock, acute respiratory failure, congestive heart failure, severe jaundice, or acute renal failure [19]. Furthermore, a systematic review showed high mortality in scrub typhus cases with central nervous system involvement or multi-organ dysfunction in pregnant women [15]. Reliable diagnostic tests would help physicians manage suspected scrub typhus cases.

Little information is available about malaria and scrub typhus co-infection. We aimed to investigate the pooled prevalence of malaria and scrub typhus co-infection among febrile patients, the prevalence of scrub typhus infection among patients with malaria, and the odds of co-infection. The data will help diagnose and manage co-infected patients in endemic areas.

## Methods

### Protocol and registration

The protocol of this systematic review was registered at PROSPERO with ID CRD42021255893. We followed the preferred reporting criteria for systematic reviews and meta-analyses (PRISMA) [21].

### Search strategy

We used three databases to search for relevant studies, PubMed, Scopus, and Web of Science. We used the keyword combination “(malaria OR plasmodium) AND scrub typhus.” The date range was from inception to May 12, 2021. The searches were unrestricted for language or year of publication. We also searched reference lists and Google Scholar to maximize studies and prevent relevant studies from being missed. The search strategy is described fully in Additional file 1: Table S1.

### Eligibility criteria

We used the PICo (P: participants, I: phenomena of interest, Co: context) or PICO (P: participants, I: phenomena of interest, C: control, O: outcome of interest) approach to select eligible studies. For the primary outcome, we used (i) P: febrile patients suspected of malaria or scrub typhus infection, (ii) I: patients with co-infection with malaria and scrub typhus, (iii) Co: none. For the secondary outcome, we used (i) P: patients with malaria infection, (ii) I: patients with co-infection of malaria and scrub typhus, (iii) Co: none. For the tertiary outcome, we used (i) P: febrile patients suspected of malaria or scrub typhus infection, (ii) I: patients with co-infection of malaria and scrub typhus, (iii) C: febrile patients without malaria and scrub typhus, and (iv) O: chance of co-infection. We detected *Plasmodium* infections by microscopy (gold standard), rapid diagnostic test (RDT), or a molecular method. We detected scrub typhus infections using indirect fluorescence assay (IFA) (gold standard), IgM enzyme-linked immunosorbent assay (ELISA), or RDT. All studies that reported co-infections with malaria and scrub typhus were considered. Case reports and case series, reviews, correspondence, animal studies, experimental studies, and test evaluation studies were excluded.

### Study selection

We reviewed all studies reporting malaria and scrub typhus co-infection. First, we excluded duplicates. Second, we screened the titles and abstracts, excluding non-related studies. Third, we read the full text and excluded irrelevant studies for various reasons. The study selection process was performed using EndNote X8 for reference management. Two authors (MK, SK) selected the studies independently and resolved any disagreements by a discussion with the third author (PW).

### Data extraction

We extracted the following information: first author’s name, year of publication, study site, year conducted, study design, age group, gender, number of participants, number of co-infections, number of malaria cases, number of scrub typhus cases, diagnostic test for malaria, and diagnostic test for scrub typhus. We recorded the data in a spreadsheet for further analysis. Two authors (MK and SK) independently extracted the data and resolved any inconsistency or disagreement by consensus.

### Quality of the included studies

We assessed the quality of the selected studies using the checklist for analytical cross-sectional studies developed by the Joanna Briggs Institute. The checklist was based on study design, study conduct, and analysis of the outcome of interest. High-quality studies received seven to eight points, while moderate-quality studies scored four to six points. Studies scoring fewer than four points were excluded. Two authors (MK and SK) assessed the included studies’ quality.

### Data analysis

The pooled prevalence of malaria and scrub typhus co-infection among febrile patients was estimated using the DerSimonian–Laird method for the random-effects model, based on the inverse variance approach for measuring weight as described previously [22, 23]. The number of co-infected patients and participants tested for both pathogens was used in the meta-analysis. The pooled prevalence of scrub typhus infection among malaria-positive patients was estimated by applying the random-effect model using the number of scrub typhus infections and malaria-positive cases. The pooled odds of co-infection were estimated using the following data: (1) the number of co-infections, (2) the number of malaria infections without scrub typhus, (3) the number of scrub typhus infections without malaria, and (4) the number of febrile patients without scrub typhus or malaria. The individual study results and the summary estimates were visualized with a forest plot as point estimates (effect size, prevalence) and 95% confidence intervals (CI). The heterogeneity of the included studies was assessed by Cochrane Q (*P* > 0.05 indicated significant heterogeneity) or *I*^2^ statistics, with *I*^2^ values < 25%, 25–75%, and > 75% interpreted as low, moderate, and high heterogeneity, respectively [24]. We analyzed the subgroup analysis of countries and diagnostic tests for scrub typhus to explore the source of heterogeneity among the included studies. Publication bias was assessed if more than 10 studies were used to estimate the outcome [25]. All data analyses were performed using Stata version 14 (StataCorp, College Station, TX, USA).

## Results

### Search results

**Fig. 1:**
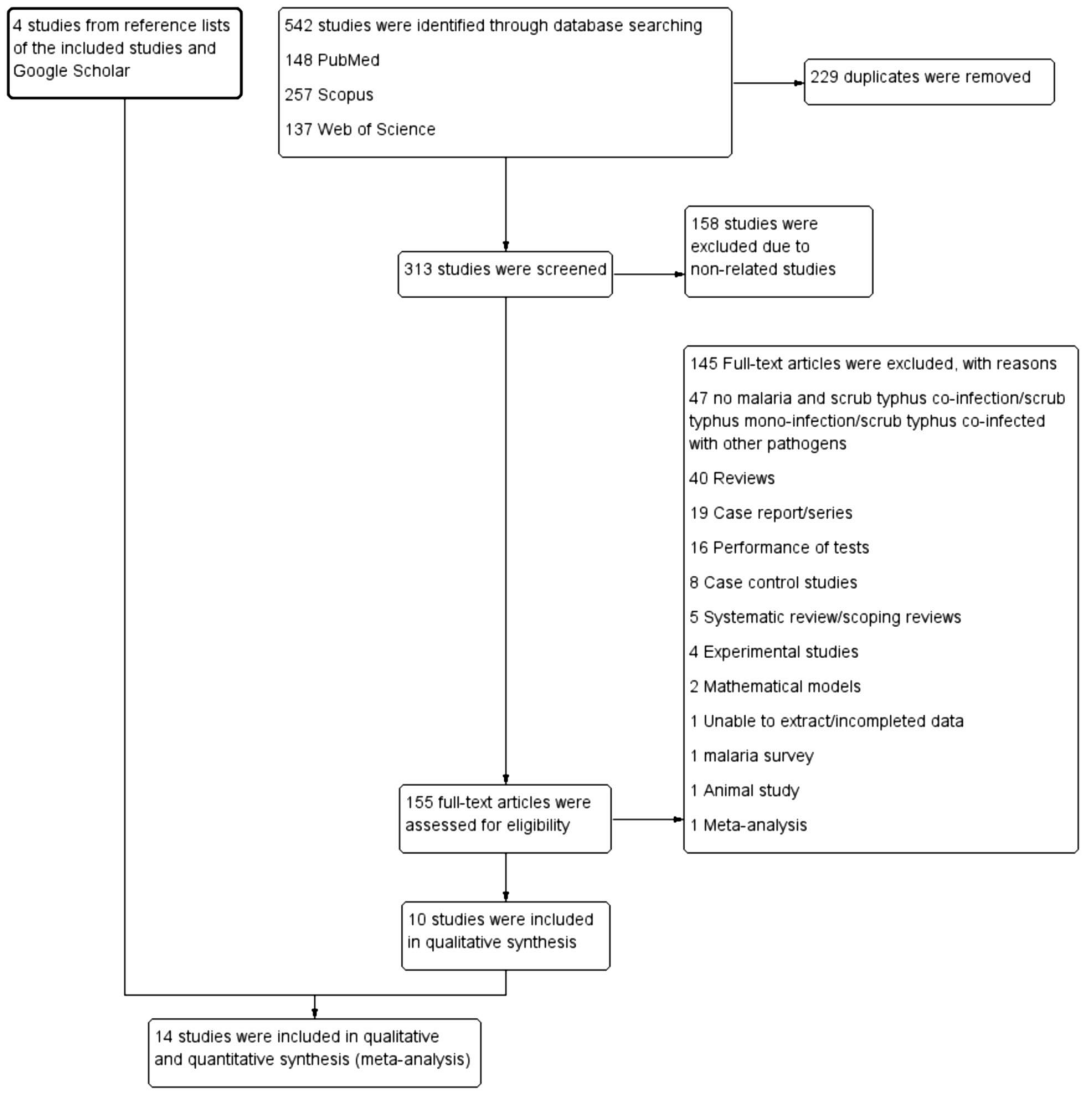
Study flow diagram

Among the 542 studies retrieved from the three databases, 10 studies [26–35] met the inclusion criteria. We found four studies [36–39] in the reference lists of the included studies and Google Scholar. Finally, 14 studies were included in this systematic review and meta-analysis (Fig. 1).

### Characteristics of the included studies

Fourteen studies that reported malaria and scrub typhus co-infections were included in the study (Table 1). All the studies were published between 1998 and 2020. Six studies (42.9%) were retrospective observational studies [26, 30, 33, 34, 36, 38], six were cross-sectional studies [27–29, 31, 37, 39], and two [32, 35] were cohort studies. Most studies (10/14, 71.4%) were conducted in India [26–28, 30–33, 37–39], and four studies [29, 34–36] were conducted in Thailand. Nine studies [26–28, 31–33, 35, 38, 39] enrolled febrile patients (7920 cases), four [29, 34, 36, 37] enrolled malaria-positive patients (639 cases), and one study [30] enrolled scrub-typhus-positive patients (240 cases). In the febrile patient studies, 56 co-infections in 7920 patients were reported, and in the studies that enrolled malaria-positive patients [29, 34, 36, 37], 265 of 639 cases were reported co-infected. Six concurrent malaria infections were reported in the study [30] that enrolled 240 scrub-typhus-positive patients. The enrolled patients were > 12 years old in seven studies [29, 30, 32, 33, 35, 38, 39], any age groups in two [29–33], and ages were not specified (NS) in two [26, 37].

**Table 1:** Characteristics of the included studies

Author	Study site	Year of study	Study design	Participants	Age	Male/female	Co-infections	All malaria cases	Test for malaria	Test for scrub typhus
Ahmad et al. [26]	India	2012–2013	Retrospective observational study	233 febrile patients	NS	NS	19	89	NS	IgM ELISA
Bal et al. [27]	India	2017	Cross-sectional study	413 febrile patients	< 15 years	124:289	6	16	RDT	IgM ELISA
Behera et al. [28]	India	2017	Cross-sectional study	432 adult (290) and pediatric patients (142)	All ages	NS	4	13	Microscopy, RDT	IgM ELISA, PCR
Chanyasanha et al. [29]	Thailand	1994	Cross-sectional study	200 malaria-positive patients	> 15 years	173:63	119	200	NS	IFA
Kotepui et al. [36]	Thailand	2013–2015	Retrospective observational study	179 malaria-positive patients	All ages	110:69	112	179	Microscopy	RDT
Mandage et al., 2020 [37]	India	2017–2018	Cross-sectional study	66 malaria-positive patients	NS	NS	5	66	Microscopy, RDT, PCR	PCR
McGready et al. [35]	Thailand	2004–2006	Cohort study	203 febrile pregnant women	Adults	0:203	2	51	Microscopy	PCR
Mittal et al. [38]	India	2012–2013	Retrospective observational study	2547 febrile pregnant women	Adults	0:2547	11	172	Microscopy, RDT	IgM ELISA
Mohanty et al. [30]	India	2016–2018	Retrospective observational study	240 scrub-typhus-positive patients	≥ 18 years	131:109	6	6	Microscopy	IgM ELISA
Mørch et al. [31]	India	2011–2012	Cross-sectional study	1564 febrile patients	≥ 5 years	895:632	8	268	Microscopy, RDT, PCR	IgM ELISA, IFA
Patil et al. [39]	India	2018–2019	Cross-sectional study	200 febrile patients	≥ 13 years	123:77	3	3	Microscopy	Weil Felix test
Raina et al. [32]	India	2016	Cohort study	1164 febrile patients	≥ 18 years	NS	2	9	Microscopy	IgM ELISA
Singh et al. [33]	India	2013	Retrospective observational study	1141 febrile patients	> 12 years	618:523	1	158	Microscopy, RDT	IgM ELISA
Singhsilarak et al. [34]	Thailand	NS	Retrospective observational study	194 malaria-positive patients	All ages	NS	29	194	Microscopy	IFA

*ELISA* enzyme-linked immunosorbent assay, *NS* not specified, *RDT* rapid diagnostic test, *IFA* immunofluorescence assay, *PCR* polymerase chain reaction

### Quality of the included studies

The quality of the included studies was assessed using the Joanna Briggs Institute checklist for analytical cross-sectional studies. Four studies [29, 31, 35, 38] were considered high-quality studies, while 10 studies [26–28, 30, 32–34, 36, 37, 39] were categorized as moderate-quality studies (Additional file 2: Table S2).

### Prevalence of malaria and scrub typhus co-infection among febrile patients

**Fig. 2:**
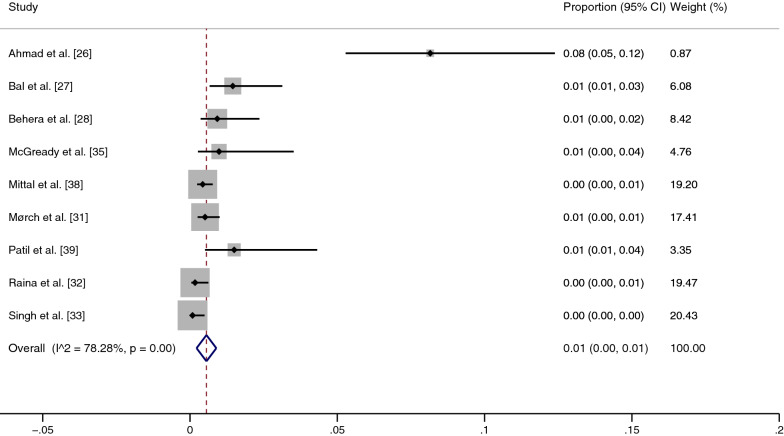
Meta-analysis for pooled prevalence of malaria and scrub typhus co-infection in febrile patients. *CI* confidence interval

The pooled prevalence of malaria and scrub typhus co-infection (56 cases) among febrile patients (7920 cases) was estimated from nine studies [26–28, 31–33, 35, 38, 39]. The highest prevalence of co-infection was demonstrated in a study by Ahmad et al. (8%) [26], while the lowest prevalence (0%) was seen in three studies [32, 33, 38]. Overall, the results showed that the pooled prevalence of malaria and scrub typhus co-infection in febrile patients was 1% (95% CI: 0–1%, *I*^2^: 78.28%) (Fig. 2).

### Prevalence of scrub typhus infections among patients with malaria

The pooled prevalence of scrub typhus infection, 321 out of 1418 patients with malaria (cases), was estimated from 13 studies [26–29, 31–39]. In India, the results of individual studies showed prevalence heterogeneity (1–100%); the highest prevalence (100%) was reported in the study by Patil et al. [39], while the lowest prevalence was found by Singh et al. [33] (1%, 95% CI: 0–3%). Overall, the results showed that the pooled prevalence of scrub typhus infection among patients with malaria in India was 8% (95% CI: 4–13%, *I*^2^: 85.87%, nine studies with 59/794 cases).

**Fig. 3:**
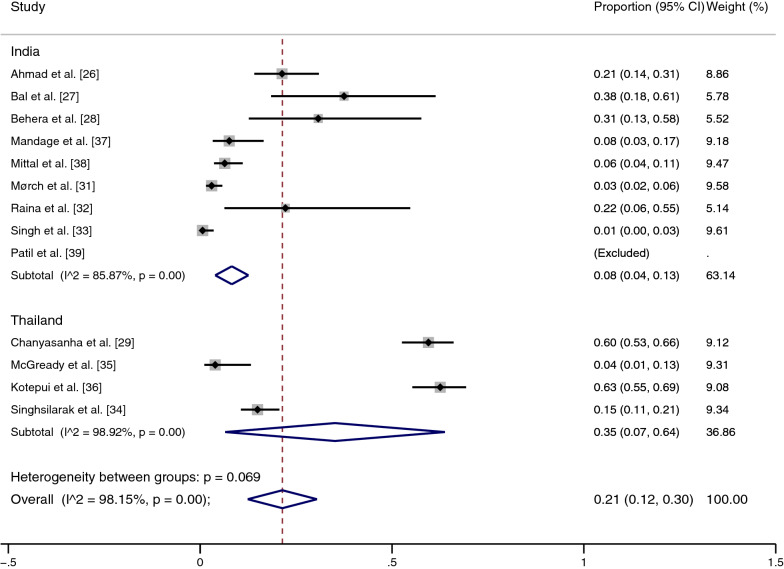
Meta-analysis for pooled prevalence of scrub typhus in patients with malaria according to country (India and Thailand). *CI* confidence interval

In Thailand, studies also showed prevalence heterogeneity (4–63%), in which the studies by Kotepui et al. [36] and Chanyasanha et al. [29] demonstrated high prevalence at 63% (95% CI: 55–69%) and 60% (95% CI: 53–66%), respectively, while the lowest prevalence was demonstrated by McGready et al. [35], with 4% (95% CI: 1–13%). Overall, the pooled prevalence of scrub typhus infection in patients with malaria in Thailand was 35% (95% CI: 7–64%, *I*^2^: 98.9%, four studies with 262/624 cases). Combined with the results of the estimated prevalence in India and Thailand, the pooled prevalence of scrub typhus infection among patients with malaria was 21% (95% CI: 12–30%, *I*^2^: 98.15%) (Fig. 3).

### Subgroup analysis by diagnostic tests

**Fig. 4:**
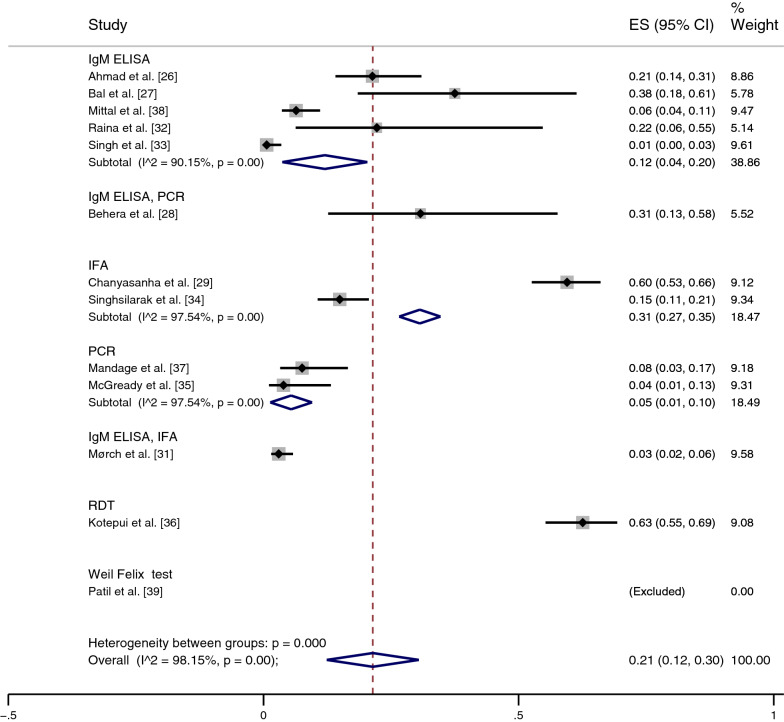
Meta-analysis for pooled prevalence of scrub typhus in patients with malaria according to diagnostic test used. *CI* confidence interval, *ELISA* enzyme-linked immunosorbent assay, *ES* estimated proportion, *IFA* indirect fluorescence assay, *PCR* polymerase chain reaction, *RDT* rapid diagnostic test

The subgroup analysis of the diagnostic tests for scrub typhus was performed using 13 studies [26–29, 31–39]. The pooled prevalence of scrub typhus infection among patients diagnosed with malaria was 12% (95% CI: 4–20%, *I*^2^: 90.15%) according to IgM ELISA alone, 31% with IgM ELISA/polymerase chain reaction (PCR) (95% CI: 13–58%), 31% with IFA alone (95% CI: 27–35%, *I*^2^: 97%), 3% with IgM ELISA/IFA (95% CI: 2–6%), 5% with PCR (95% CI: 1–10%), 63% with RDT (95% CI: 55–69%), and 100% with the Weil Felix test (Fig. 4).

### Odds of co-infection

**Fig. 5:**
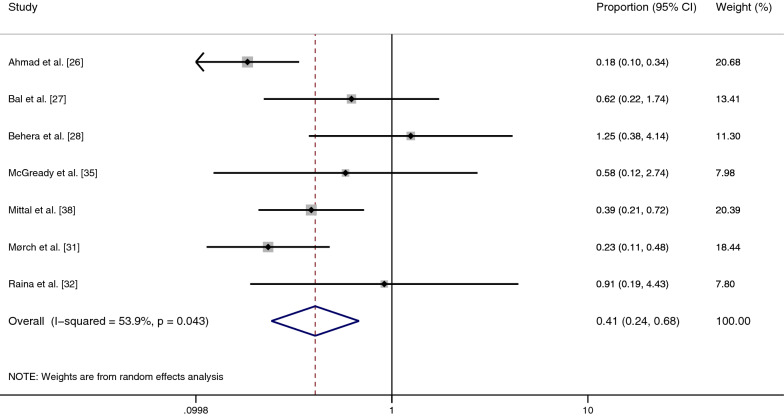
Meta-analysis for odds of malaria and scrub typhus co-infection in febrile patients. *CI* confidence interval

The pooled odds of co-infection were estimated using seven studies [26–28, 31, 32, 35, 38]. Three studies [26, 31, 38] demonstrated lower odds of co-infection, but four studies [27, 28, 32, 35] showed no difference in the odds of co-infection. Overall, the results showed that malaria and scrub typhus co-infection did not occur by chance (*P* = 0.001, odds: 0.41, 95% CI: 0.24–0.68%, *I*^2^: 53.9%) (Fig. 5).

### Sensitivity analysis

**Fig. 6:**
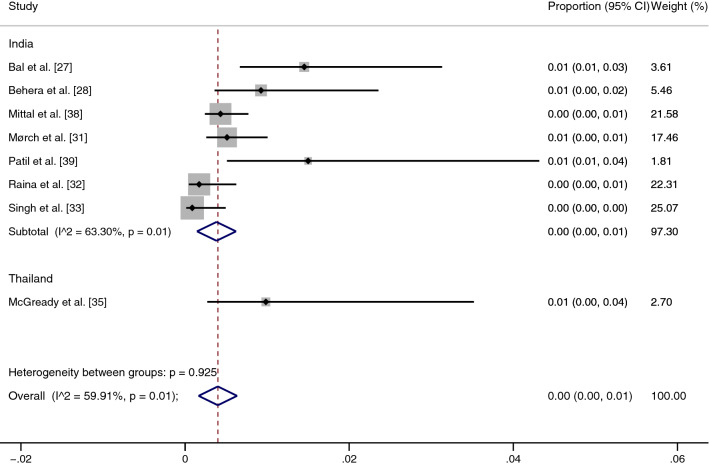
Sensitivity test of the pooled prevalence of malaria and scrub typhus co-infection in febrile patients when the outlier was excluded. *CI* confidence interval

**Fig. 7:**
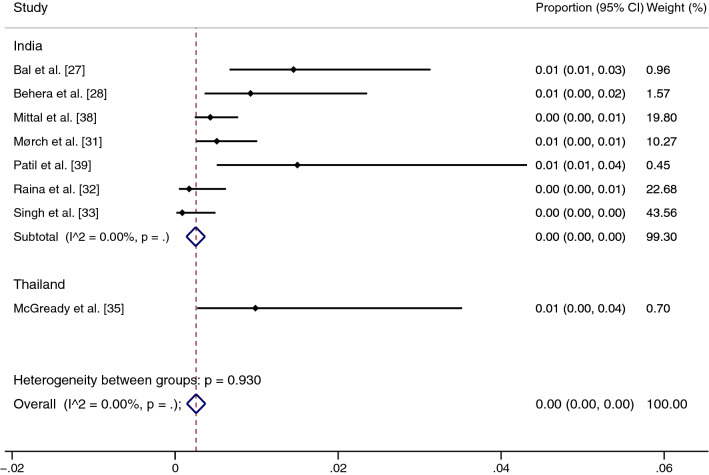
Sensitivity test of the pooled prevalence of scrub typhus in patients with malaria when the fixed-effect model was used. *CI* confidence interval

Because the pooled prevalence of malaria and scrub typhus co-infection among febrile patients was heterogeneous, we used the leave-one-out method [40] for statistical validity and homogeneity of the results. Ahmad et al.'s [26] study in India, with the highest prevalence, was excluded as an outlier. The sensitivity analysis showed that the pooled prevalence of malaria and scrub typhus co-infection among febrile patients was very low (0%, 95% CI: 0–1%, *I*^2^: 59.91%) (Fig. 6). The fixed-effect model showed that the pooled prevalence of malaria and scrub typhus co-infection among febrile patients was 0% (95% CI: 0–0%, *I*^2^: 0%) in India and 1% (95% CI: 0–4%, *I*^2^: 0%) in Thailand (Fig. 7).

### Publication bias

**Fig. 8:**
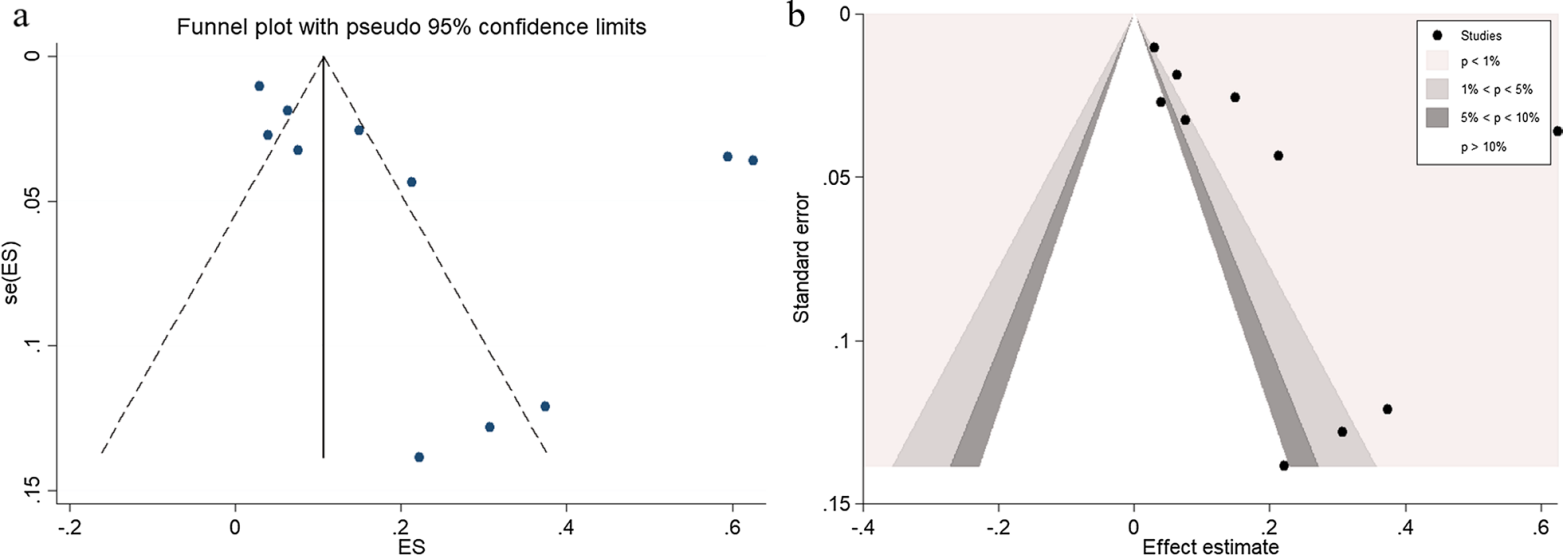
Funnel plot (**a**) and contour-enhanced funnel plot (**b**)

Publication bias was assessed by a funnel plot using the effect size (ES, pooled prevalence) and standard error of the ES (seES) from the 12 studies [26–29, 31–38] that assessed the pooled prevalence of scrub typhus infection in patients with malaria. The results showed the asymmetric distribution of the ES (Fig. 8a). The Egger’s test showed that small study effects occurred (*P* = 0.023, coefficient: 6.55, standard error: 2.43, *t*: 2.69). Further contour-enhanced funnel plot analysis showed that most of the studies missing from non-significant areas (*P* > 0.01) indicated that the cause of funnel plot asymmetry was likely to be due to publication bias (Fig. 8b).

## Discussion

Malaria and scrub typhus are vector-borne diseases transmitted by different arthropod vectors [41, 42]. The meta-analysis in this study showed a low prevalence (0–1%) of malaria and scrub typhus co-infection among febrile patients, and these types of infections were limited to India. Although there was heterogeneity of prevalence in the studies included in the meta-analysis (78%), four studies conducted in India demonstrated the same prevalence (1%). The study by Ahmad et al. (8%) [26], which differed, used IgM ELISA to detect scrub typhus IgM antibodies. It is unknown whether malaria infection can induce IgM antibodies that cross-react with other fevers. A report suggested that an apparent high co-infection rate might be caused by cross-reactivity and the background positivity of patients in endemic areas rather than actual co-infection [31]. Therefore, the results of a single serological test for scrub typhus diagnosis should be interpreted cautiously if malaria is endemic in the same area. A previous study showed that malaria and scrub typhus co-infection reduced hepatosplenomegaly or other organ dysfunctions, compared with malaria and scrub typhus mono-infections. However, only small groups of patients were investigated [26]. The mechanisms of protection against severe disease are not well understood, but a recent study conducted in India showed that malaria and scrub typhus co-infection is associated with severe malaria (adjusted OR: 1.1, 95% CI: 0.1–7.8%) [37]. This suggests that this co-infection might be like malaria and dengue co-infection in its impact on disease severity, leading to severe malaria or severe dengue [43]. However, the co-infection prevalence data for these pathogens are limited. Further research is needed to determine whether co-infecting pathogens can impact disease outcomes.

Our meta-analysis showed a high prevalence of scrub typhus infections among patients with malaria in Thailand (35%). This was associated with high heterogeneity (98.9%), possibly caused by differences in the study participants, scrub typhus diagnostic tests, study area, or the year of the studies. In the study by Kotepui et al. [36], RDT was used to detect scrub typhus IgM/IgG. The manufacturer claims it provides 100% sensitivity, 100% specificity, and 100% accuracy (Rickettsia IgG/IgM Combo Test, LumiQuick Diagnostics, Inc., USA). The study design involved retrospective collection of the data of patients who had been previously infected with malaria and tested for scrub typhus; therefore, the prevalence of scrub typhus infections in malaria patients may have been overestimated. Chanyasanha et al. [29] demonstrated a prevalence as high as that reported by Kotepui et al. [36]. In contrast, a study of pregnant women who attended antenatal care by Mittal et al. [38] showed a low prevalence of scrub typhus infections among patients with malaria (6%). The difference in prevalence might be due to the high prevalence of scrub typhus before the year 2000, when, as the study by Chanyasanha et al. [29] showed, there was a prevalence of 59.5% among malaria clinic patients. After 2000, the scrub typhus prevalence was 9.1–11.1% [44]. Retrospective diagnosis with RDT showed a similar performance to ELISA in Indian patients, and the RDT diagnosis of scrub typhus was more sensitive than standard IFA in acute-phase specimens [45]. Nevertheless, a recent meta-analysis showed the pooled sensitivity and specificity of commercially available RDTs were 66.0% (95% CI 0.37–0.86%) and 92.0% (95% CI 0.83–0.97%), respectively, with a high degree of heterogeneity between the reviewed studies [46]. The performance of RDTs makes them a good choice for the early diagnosis of scrub typhus in remote or rural regions of endemic countries.

In India, a meta-analysis demonstrated a low prevalence of malaria and scrub typhus co-infection in febrile patients and a low prevalence of scrub typhus infection in malaria patients. There was heterogeneity in the prevalence of malaria and scrub typhus co-infection in febrile patients among the studies conducted in India (0–1%). However, the prevalence of co-infection tended toward homogeneity when the meta-analysis excluded the results of Ahmad et al. [26]. The low prevalence of malaria and scrub typhus co-infection among febrile patients might be because it is understudied or underreported, as suggested by Mørch et al. [31]. Epidemiological studies have reported that socioeconomic status and occupation were related to the risk of scrub typhus [47, 48]. Scrub typhus prevalence is ~ 3–9% in Vietnam [49, 50], 6–23% in Bhutan [51, 52], and 4–5% in Malaysia [53, 54]. In addition, scrub typhus was reported in China, Japan, South Korea, Indonesia, the Philippines, and Australia [55, 56]. However, malaria and scrub typhus co-infections only occurred in Thailand and India; the reasons for this are poorly understood. Possibly, physicians in countries other than Thailand and India did not investigate febrile patients for scrub typhus, as there is no Global Fund support for this, unlike with acquired immunodeficiency syndrome, tuberculosis, and malaria [57]. Also, some febrile patients may self-medicate with drugs like doxycycline, which is an anti-rickettsia drug and can lead to the misdiagnosis of scrub typhus. Therefore, the underreporting of scrub typhus in febrile or malaria patients is likely.

The present meta-analysis showed significantly decreased odds of co-infection, which indicates that malaria and scrub typhus co-infections occurred by chance rather than infection by one pathogen leading to an increased risk of contracting the other infection. However, co-infection risk was demonstrated in only two studies [26, 31], while other studies [27, 28, 32, 35] reported no difference in the odds of co-infection. The association between malaria and scrub typhus might be caused by the large geographic overlap in their distribution or the high prevalence of previous infections leading to cross-reactivity and subclinical infections rather than a high prevalence of co-infections. In addition, scrub typhus infections may prevent malaria or reduce its clinical presentation, but this has not yet been investigated, and further studies are needed.

The present study had limitations. First, only a small number of studies in Thailand and India reported malaria and scrub typhus co-infections. Therefore, the low prevalence of co-infection among febrile patients might be due to the underreporting of scrub typhus or misdiagnosis. Second, the high heterogeneity of measurements among the studies might have influenced the interpretation of the meta-analysis. Therefore, the pooled prevalence must be interpreted in consideration of the results of individual studies and sensitivity tests.

## Conclusion

The findings of this study emphasize the importance of further research on malaria and scrub typhus co-infection in endemic areas. The outcomes also highlight the importance of interpreting diagnostic tests together with clinical signs and symptoms to facilitate the precise management of febrile patients who live in areas endemic for both diseases.

## Supplementary Information

**Additional file 1: Table S1.** Search terms.

**Additional file 2: Table S2.** Quality of the included studies.

## Data Availability

All data relating to the present study in this manuscript are available.

## Abbreviations

CI: Confidence intervals
ELISA: Enzyme-linked immunosorbent assay
IFA: Indirect fluorescence assay
IQR: Interquartile range
NS: Not specified
PCR: Polymerase chain reaction
PRISMA: Preferred reporting criteria for systematic reviews and meta-analyses
RDT: Rapid diagnostic test
WHO: World Health Organization
