# Supplementary material for: Prevalence of malaria and scrub typhus co-infection in febrile patients: a systematic review and meta-analysis
Source: Parasit Vectors. 2021 Sep 14;14:471. doi: 10.1186/s13071-021-04969-y (PMC8442375; doi:10.1186/s13071-021-04969-y)
Supplement: Supplementary file 1 — Additional file 1: Table S1. Search terms. [file 13071_2021_4969_MOESM1_ESM.docx]

**Prevalence of malaria and scrub typhus co-infection in febrile patients: a systematic review and meta-analysis**

Polrat Wilairatana^1^, Saruda Kuraeiad^2^, Pongruj Rattaprasert^3^, Manas Kotepui^2*^

^1^Department of Clinical Tropical Medicine, Faculty of Tropical Medicine, Mahidol University, Bangkok, Thailand

^2^Medical Technology, School of Allied Health Sciences, Walailak University, Tha Sala, Nakhon Si Thammarat, Thailand

^3^Department of Protozoology, Faculty of Tropical Medicine, Mahidol University, Bangkok, Thailand

^*^Corresponding author

Manas Kotepui; [manas.ko@wu.ac.th](mailto:manas.ko@wu.ac.th), Tel.: +66954392469

Polrat Wilairatana; [polrat.wil@mahidol.ac.th](mailto:polrat.wil@mahidol.ac.th)

Saruda Kuraeiad; [saruda.ku@wu.ac.th](mailto:saruda.ku@wu.ac.th)

Pongruj Rattaprasert; pongruj.rat@mahidol.ac.th

**Table S1. Search term**

| **Databases** | **Search terms** | **Date** |
| --- | --- | --- |
| MEDLINE | (malaria OR plasmodium) AND “scrub typhus”  Search results: 148 | 12 May 2021 |
| Scopus | (malaria OR plasmodium) AND “scrub typhus”  Search option: Title, abstract, keywords  Search results: 257 | 12 May 2021 |
| ISI Web of Science | (malaria OR plasmodium) AND “scrub typhus”  Search option: All fields  Search results: 137 | 12 May 2021 |
