# Supplementary material for: Prevalence of malaria and scrub typhus co-infection in febrile patients: a systematic review and meta-analysis
Source: Parasit Vectors. 2021 Sep 14;14:471. doi: 10.1186/s13071-021-04969-y (PMC8442375; doi:10.1186/s13071-021-04969-y)
Supplement: Supplementary file 2 — Additional file 2: Table S2. Quality of the included studies. [file 13071_2021_4969_MOESM2_ESM.docx]

**Prevalence of malaria and scrub typhus co-infection in febrile patients: a systematic review and meta-analysis**

Polrat Wilairatana^1^, Saruda Kuraeiad^2^, Pongruj Rattaprasert^3^, Manas Kotepui^2*^

^1^Department of Clinical Tropical Medicine, Faculty of Tropical Medicine, Mahidol University, Bangkok, Thailand

^2^Medical Technology, School of Allied Health Sciences, Walailak University, Tha Sala, Nakhon Si Thammarat, Thailand

^3^Department of Protozoology, Faculty of Tropical Medicine, Mahidol University, Bangkok, Thailand

^*^Corresponding author

Manas Kotepui; [manas.ko@wu.ac.th](mailto:manas.ko@wu.ac.th), Tel.: +66954392469

Polrat Wilairatana; [polrat.wil@mahidol.ac.th](mailto:polrat.wil@mahidol.ac.th)

Saruda Kuraeiad; [saruda.ku@wu.ac.th](mailto:saruda.ku@wu.ac.th)

Pongruj Rattaprasert; pongruj.rat@mahidol.ac.th

| No. | Authors | Eligibility criteria | Study subjects and the setting | Exposure measured in a valid and reliable way 'gold standard' | A specified diagnosis or definition | Confounding factors | Dealing with confounding factors | Outcomes measured in a valid and reliable way | Appropriate statistical analysis | Scores (8) | Quality (high, moderate, low) |
| --- | --- | --- | --- | --- | --- | --- | --- | --- | --- | --- | --- |
| 1 | Ahmad et al., 2016 | Yes | Yes | No | Yes | No | NA | Yes | Yes | 6 | Moderate |
| 2 | Bal et al., 2019 | Yes | Yes | No | Yes | No | NA | Yes | Yes | 6 | Moderate |
| 3 | Behera et al., 2019 | Yes | Yes | No | Yes | No | NA | Yes | Yes | 6 | Moderate |
| 4 | Chanyasanha et al., 1998 | Yes | Yes | Yes | Yes | No | NA | Yes | Yes | 7 | High |
| 5 | Mandage et al., 2020 | Yes | No | Yes | Yes | No | NA | Yes | Yes | 6 | Moderate |
| 6 | McGready et al., 2010 | Yes | Yes | Yes | Yes | No | NA | Yes | Yes | 7 | High |
| 7 | Mittal et al., 2015 | Yes | Yes | Yes | Yes | No | NA | Yes | Yes | 7 | High |
| 8 | Mohanty et al., 2020 | Yes | Yes | No | Yes | No | NA | Yes | Yes | 6 | Moderate |
| 9 | Mørch et al., 2017 | Yes | Yes | Yes | Yes | No | NA | Yes | Yes | 7 | High |
| 10 | Kotepui et al., 2017 | Yes | Yes | No | Yes | No | NA | Yes | Yes | 6 | Moderate |
| 11 | Patil et al., 2019 | Yes | Yes | No | Yes | No | NA | Yes | Yes | 6 | Moderate |
| 12 | Raina et al., 2018 | Yes | Yes | No | Yes | No | NA | Yes | Yes | 6 | Moderate |
| 13 | Singh et al., 2014 | Yes | Yes | No | Yes | No | NA | Yes | Yes | 6 | Moderate |
| 14 | Singhsilarak et al., 2006 | Yes | No | Yes | Yes | No | NA | Yes | Yes | 6 | Moderate |

NA, Not Applicable
